# LYZL6, an acidic, bacteriolytic, human sperm-related protein, plays a role in fertilization

**DOI:** 10.1371/journal.pone.0171452

**Published:** 2017-02-09

**Authors:** Peng Huang, Wenshu Li, Zhifang Yang, Ning Zhang, Yixin Xu, Jianying Bao, Deke Jiang, Xianping Dong

**Affiliations:** 1 College of Basic Medicine, Shanghai University of Medicine and Health Sciences, Shanghai, People’s Republic of China; 2 College of Arts and Sciences, New York University, Shanghai, People’s Republic of China; 3 College of Pharmacy, Shanghai University of Medicine and Health Sciences, Shanghai, People’s Republic of China; 4 State Key Laboratory of Organ Failure Research, Southern Medical University, Guangzhou, People’s Republic of China; 5 Department of Physiology and Biophysics, Dalhousie University, Halifax, Canada; Kermanshah University of Medical Sciences, ISLAMIC REPUBLIC OF IRAN

## Abstract

Lysozyme-like proteins (LYZLs) belong to the c-type lysozyme/α-lactalbumin family and are selectively expressed in the mammalian male reproductive tract. Two members, human sperm lysozyme-like protein (SLLP) -1 and mouse LYZL4, have been reported to contribute to fertilization but show no bacteriolytic activity. Here, we focused on the possible contribution of LYZL6 to immunity and fertilization. In humans, LYZL6 was selectively expressed by the testis and epididymis and became concentrated on spermatozoa. Native LYZL6 isolated from sperm extracts exhibited bacteriolytic activity against *Micrococcus lysodeikticus*. Recombinant LYZL6 (rLYZL6) reached its peak activity at pH 5.6 and 15 mM of Na^+^, and could inhibit the growth of Gram-positive, but not Gram-negative bacteria. Nevertheless, the bacteriolytic activity of rLYZL6 proved to be much lower than that of human lysozyme under physiological conditions. Immunodetection with a specific antiserum localized the LYZL6 protein on the postacrosomal membrane of mature spermatozoa. Immunoneutralization of LYZL6 significantly decreased the numbers of human spermatozoa fused with zona-free hamster eggs in a dose-dependent manner *in vitro*. Thus, we report here for the first time that LYZL6, an acidic, bacteriolytic and human sperm-related protein, is likely important for fertilization but not for the innate immunity of the male reproductive tract.

## Introduction

Lysozymes are ubiquitous hydrolases present in numerous phylogenetically diverse organisms that can catalyze the hydrolysis of β-1,4-linked glycosidic bonds between N-acetylmuramic acid and N-acetylglucosamine in the peptidoglycans of bacterial cell walls, and thereby cause bacteriolysis [1, 2]. Hence, lysozymes play important roles in protecting the host against pathogenic infections. On the base of the amino acid sequence and biological origin, lysozyme has been classified into six types: chicken-type (c-type), goose-type (g-type), invertebrate-type (i-type), and phage, bacterial, and plant lysozymes [3]. Among them, c-type lysozyme was found to differ from other types of lysozymes in molecular weight, amino acid composition, and enzymatic properties, indicating that these enzymes were the products of different genes. In mammals, the c-type lysozymes/α-lactalbumin family includes c-type lysozyme and several LYZLs which have been studied extensively [4–6].

LYZLs exhibit specific expression patterns and functions different from c-type lysozymes. For example, a high level of LYZL4 protein was found on the acrosomal region and principal piece of mouse spermatozoa. Immunoneutralization of mouse LYZL4 with its specific antibody significantly decreased *in vitro* fertilization percentages in a dose-dependent manner, implying a role in mouse fertilization [7]. In humans, genes encoding four c-type LYZLs (*LYZL2*, *LYZL4*, *LYZL6*, and *SPACA3*) have been cloned from the male reproductive tract, and all of them seem to be expressed in the testis or epididymis [6]. Among these LYZLs, the human *SPACA3* gene encodes SLLP1, antisera to which can block sperm–egg binding in a zona-free hamster egg penetration test (HEPT) [8]. Subsequently, an egg-specific membrane metalloproteinase, SAS1B (sperm acrosomal SLLP1 binding), was identified from mouse egg lysates as an SLLP1 binding partner, confirming its involvement in fertilization [9]. As to LYZL6, its actual function has not been well characterized, despite certain bacteriolytic activity of the recombinant protein being detected [10, 11].

In the present study, we report that LYZL6 is expressed in the human testis, epididymis and spermatozoa, with high enrichment in the postacrosomal region of human spermatozoa. We also show that LYZL6 exhibits bacteriolytic activity against *M*. *lysodeikticus* in a pH- and Na^+^-dependent manner and rLYZL6 protein shows weak bacteriolytic activity against Gram-positive bacteria at physiological pH. Additionally, anti-LYZL6 serum decreases the numbers of spermatozoa fused per hamster egg *in vitro*. These data suggest that LYZL6 is likely important for fertilization but not for the innate immunity of the male reproductive tract.

## Materials and methods

### Animal and semen samples

The animal experimentation was approved by the Animal Care and Use Committee of Tongji University (Shanghai, P. R. China). Two female New Zealand white rabbits (age: 2 months, weight: 2.5 kg, Songlian Laboratory Animal Center, Shanghai, P. R. China) and twenty female hamsters (age: 8 weeks, weight: 130 g, Songlian Laboratory Animal Center) were involved in the study. The animals were housed in conventional cages with free access to standard laboratory food and water at 20–24°C with a 12 h light/dark cycle. Intravenous injection of sodium pentobarbital (100 mg/kg) was used for the euthanasia of rabbits after antiserum preparation. Cervical dislocation was used for the euthanasia of hamsters prior to the collection of hamster eggs. Normal human epididymis tissue used to prepare the cDNA library was obtained from patients undergoing therapeutic epididymectomy for metastatic prostate carcinoma at Shuguang Hospital (Shanghai, P. R. China). Freshly ejaculated semen samples were collected from healthy donors with normal semen parameters according to the World Health Organization (WHO, 2010) criteria at an outpatient fertility clinic of Shuguang Hospital. Semen was obtained by masturbation after 2–3 days of sexual abstinence. Written consent was obtained by the donors for the use of semen samples. This study was approved by the Ethics Committee at Shuguang Hospital.

### Experimental design

In the present study, his-tagged LYZL6 was expressed in *E*.*coli* and used to prepare anti-LYZL6 serum. Native LYZL6 was isolated from human semen and its bacteriolytic activity *in vitro* was tested. Enzymatic properties and bacteriolytic activity of innate rLYZL6, secreted by the *Pichia pastoris* expression system, were also determined with human lysozyme (LYZ) as a control. Furthermore, the subcellular localization of LYZL6 on spermatozoa and its binding to the plasma membrane of zona-free hamster eggs were investigated respectively. Finally, its possible biological role in fertilization was assessed using the HEPT.

### Reverse-transcription polymerase chain reaction (RT–PCR)

RT-PCR was performed as described [12]. The cDNAs included in the Clontech Multiple Tissue cDNA Panels (Takara Bio Inc., Shiga, Japan) were used as PCR templates. Epididymal cDNA was prepared by our laboratory with epididymis completely cleaned of luminal contents. The LYZL6 sequence was used to design forward and reverse oligonucleotide primers for tissue expression screening. Primers (Table 1) spanned a 1300 bp intron, to give 296 bp of cDNA and 1596 bp of genomic DNA. Glyceraldehyde 3-phosphate dehydrogenase (G3PDH) was used as an internal control. All primers were manufactured by Qiagen (Hilden, Germany). Cycling conditions were: 94°C for 5 min, followed by 35 cycles of 94°C for 30 s, 65°C for 45 s, 72°C for 30 s and a final stage of 72°C for 5 min.

**Table 1:** Gene specific primers used in this study.

Gene	Direction	Sequence
*LYZL6*	Forward	5′-AAATAGATGAGTAGCGCCTTTGTC-3′
	Reverse	5′- CTTGACAGTCTACGTGGCAAAGGTTTTC -3′
*G3PDH*	Forward	5′- ACCTGACCTGCCGTCTAGAA-3′
	Reverse	5′- TCCACCACCCTGTTGCTGTA-3′

### Antiserum preparation

The anti-LYZL6 serum was prepared as previously described [13]. Based on *LYZL6* mRNA sequence (NM_001199951), we designed the following primers to amplify a fragment of cDNA in human testis library: 5′-GCAGCTTTCTTGCCCTAAATCAGGCC-3′ (forward); 5′-GACTCCACGGTGCACCCGCACCCTGTT-3′ (reverse). The polymerase chain reaction product was cloned into a pET32a expression vector (Qiagen). *Escherichia coli* BL21 was transformed with the resulting plasmid according to the supplier’s instructions. Fusion protein expression was induced with 1 mM isopropyl-β-d-thiogalactopyranoside (Qiagen) for 3 h at 37°C. Bacterial lysates were incubated with nickel–nitrilotriacetic acid–agarose (Qiagen) for 1 h to allow binding of the His-tagged LYZL6 to the resin, and then transferred to a His-binding Ni^2+^ chelation affinity column, washed and eluted according to the manufacturer’s recommendations. Fractions were analyzed on 15% gradient polyacrylamide Tris–Tricine gels and the identity of the protein was confirmed by western blotting using an anti-His-tag antibody. Two rabbits were immunized four times at intervals of 3 weeks. For the first time, rabbits were injected subcutaneously at two to four different sites with the antigen (500 μg/rabbit) in complete Freund’s adjuvant. Three booster injections were given with the same amount of protein in incomplete Freund’s adjuvant. Antiserum was harvested 1 week after the last boost and the specificity was assayed by western blotting. Cross reactivity of antiserum was checked with recombinant His-tagged LYZL4 expressed in *E*.*coli* and human milk LYZ (Sigma-Aldrich, St Louis, MO, USA).

### Preparation of sperm extracts

Freshly ejaculated semen samples were allowed to liquefy at 37°C for 1 h. Spermatozoa were purified using the ISolate^®^ kit (Irvine Scientific, Santa Ana, CA, USA) followed by centrifugation for 15 min at 400 *g*. The pellet was resuspended in PBS, centrifuged for 10 min at 400 *g* and solubilized in RIPA lysis buffer in presence of complete protease inhibitors (Santa Cruz Biotechnology, Dallas, TX, USA) at a final concentration of 9 × 10^7^ cell/mL for 30 min at 4°C. The insoluble material was pelleted by centrifugation at 10 000 g for 10 min, and the supernatant was used as the loading sample for sodium dodecyl sulfate polyacrylamide gel electrophoresis (SDS–PAGE) gels.

### Immunoblotting

For anti-serum specificity analysis, His-tagged LYZL6, His-tagged LYZL4 prepared by our laboratory and LYZ were used. Proteins and samples of human tissue extracts (Santa Cruz Biotechnology), sperm extracts and seminal plasma were loaded and separated on 12% SDS-PAGE gels, and transferred onto polyvinylidene fluoride membranes using a semi-dry electroblotter. The membranes were blocked with Tris-HCl buffer (pH 7.6) containing 0.05% (v/v) Tween-20 and 5% (w/v) non-fat powdered milk for 1 h at room temperature, and then incubated overnight at 4°C with anti-LYZL6 serum (1:1000), anti-β-actin (1:10,000; Takara Bio Inc.), and anti-LYZ serum (1:1000) prepared by our laboratory. After washing, membranes were incubated with horseradish peroxidase (HRP)-conjugated goat anti-rabbit secondary antibody (1:2000; Santa Cruz Biotechnology). HRP activity was detected using an enhanced chemiluminescence detection system (Beyotime, Shanghai, P. R. China).

### Isolation of native LYZL6

Seventeen fresh ejaculates were collected and spermatozoa were extracted as described above, followed by 1:50 dilution in phosphate buffered saline (PBS, pH 7.4). LYZL6 from sperm extracts was isolated using chitin affinity chromatography in combination with anion exchange chromatography. Briefly, chitin affinity matrix, prepared by our laboratory [10], was added to sperm extracts at a ratio of 1: 30 (v/v). The mixture was gently stirred for 30 min and precipitated for 5 min. After three washes with PBS, matrix was packed into an opened column and the bound proteins were eluted with 0.01 M acetic acid. The peak was collected and dialyzed at 4°C against 2 liters of 0.02 M Tris-HCl buffer (pH 8.0). To perform anion exchange chromatography, the sample solution was loaded onto a HiTrap Q column (7 × 25 mm, GE Healthcare, Uppsala, Sweden) run on an ÄKTA fast protein liquid chromatography system (GE Healthcare). The bound proteins were eluted with a linear gradient of 0.15 to 1 M NaCl at a flow rate of 0.5 mL/min. The peaks were collected, concentrated and lyophilized. The resulting powder was reconstituted in PBS for further analysis.

### MS identification of LYZL6

Protein samples were analyzed by SDS–PAGE and the resulting gel was stained overnight with 0.01%R-250 Coomassie Blue (Bio-Rad, Hercules, CA) in 10% acetic acid. Protein staining bands were excised,digested with trypsin, and analyzed by matrix-assisted laser desorption/ionization time-of-flight (MALDI/TOF). MALDI/TOF and MALDI/TOF/TOF were performed on an AB 4700 TOF-TOF Proteomics Analyzer (Applied Biosystems, Framingham, MA) as described previously [12]. The resulting peptide peaks were searched against the MSDB and NCBI databases using the MASCOT search engine.

### Radial diffusion assay

Bacteriolytic activity of native LYZL6 was tested by the agar-based diffusion assay against one gram-positive strain (*M*. *lysodeikticus*) and one gram-negative strain (*Escherichia coli*). Briefly, One hundred microliters of the bacterial suspension (4 × 10^7^ cell/mL) was mixed with 12 mL of molten underlay solution and poured into 90 mm Petri dishes. The underlay solution consisted of 1% low melting-point agarose (Sigma-Aldrich) and a 1:100 dilution of tryptone soya broth (TSB) in PBS (pH 6.0). 5 mm-diameter wells were punched in the solidified gel, and 10 μL of the solution of native LYZL6 isolated from semen was applied to the gel. Plates were incubated at 37°C for 3 h to allow the protein to diffuse. The underlay was then covered with 12 mL of molten overlay solution which consisted of 6% TSB and 1% agarose in PBS. The overlay was allowed to cool and solidify and the plates were incubated overnight at 37°C.

### Production of recombinant LYZL6 in *P*. *pastoris*

The *LYZL6* gene was synthesized according to the codon usage preference of *P*. *pastoris* [13] and cloned into the pCR2.1-TOPOR vector (Takara Bio Inc.). The pCR2.1-TOPOR/*LYZL6* plasmid was digested with *EcoR I* and *Not I* restriction enzymes and then cloned into the *EcoR I*/*Not I* site of the *Pichia* expression vector pPIC9K. The resulting plasmid was transformed into *E*. *coli* DH5α for nucleotide sequencing to ensure in-frame insertion. The constructed pPIC9K/*LYZL6* plasmid was linearized by *Sac I* and transformed into competent cells of *P*. *pastoris* GS115 by electroporation. The transformed cells were screened on yeast extract peptone dextrose (YPD) plates supplemented with increasing Geneticin^®^ (G418) concentrations. A clone from a 4 mg/mL G418 YPD plate was inoculated in 2 mL YPD medium for ~48 h at 30°C, and then transferred into 0.5 L buffered minimal glycerol-complex medium and cultured for ~48 h at 30°C. To induce expression, methanol was added at a final concentration of 1% every 24 h. After 3 days of induction, the supernatant was collected by centrifugation. Recombinant LYZL6 (rLYZL6) was purified by chitin affinity chromatography. The pooled fractions was lyophilized, reconstituted in PBS and subjected to purity analysis.

### Characterization of enzymatic properties

Enzymatic characterization was performed as described [12]. A suspension of *M*. *lysodeikticus* was used as a substrate and bacteriolytic activity was determined according to a turbidimetric assay. To determine the optimal pH, substrate suspensions of 5 mM Na-acetate and 5 mM Na-phosphate buffers were used for pH ranges of 3.6–5.2 and 6.0–9.2, respectively. To evaluate the salt sensitivity, the final ion concentration of the substrate suspension was adjusted with NaCl. For all of the assays, a 100-μL portion of rLYZL6 solution (50 μg/mL) was added to a 2.5 mL substrate suspension and identical amounts of LYZ were applied as controls.

### Assessment of bacteriolytic activity

Bacteriolytic activity of rLYZL6 was assessed using the colony-forming unit (CFU) assay. Three Gram-positive strains (*M*. *lysodeikticus*, *Bacillus subtilis* and *Staphylococcus aureus*) and two Gram-negative strains (*Pseudomonas aeruginosa* and *E*. *coli*) were used as substrates. Briefly, single colonies of bacteria were inoculated in Luria–Bertani (LB) broth overnight in a shaking incubator at 37°C and then diluted 1:50 with LB broth and grown until the mid-logarithmic phase was reached. After centrifugation, bacterial pellets were washed twice and resuspended in 1% peptone broth with pH 5.6 or 7.4 to give a density of 2 × 10^5^ cell/mL. One hundred microliters of the bacterial suspension was mixed with an equal volume of the solution of rLYZL6 (50 μg/mL) or LYZ (50 μg/mL). The mixture was incubated at 37°C for 2 h, and then 100 μL aliquots were plated onto LB agar. Plates were incubated at 37°C until distinct colonies could be counted. The bacteriolytic activity was expressed as the percentage of colonies surviving [survival % = (number of colonies after treatment with rLYZL6/number of colonies surviving without rLYZL6) × 100]. Distilled water was used as a control and all assays were performed in triplicate.

### Immunofluorescence of spermatozoa

Immunofluorescent staining was carried out on live human spermatozoa. Freshly ejaculated spermatozoa were purified as described above and resuspended in PBS. After two washes, 15-μL droplets were smeared on polylysine-coated slides and left to air-dry for 15 min. In some experiments, the cells were fixed with 4% paraformaldehyde solution for 10 min and permeabilized with methanol for 30 min. Nonspecific binding was blocked with PBS containing 3% (w/v) bovine serum albumin (BSA) for 10 min. After two washes, the slides were incubated in 50 μL anti-LYZL6 serum (1:100 dilution) or preimmune rabbit serum (1:100 dilution) overnight at 4°C. The slides were washed three times with PBS and rhodamine-conjugated goat anti-rabbit IgG Alexa Fluor 568 (Sigma-Aldrich) was applied in 50 μL (1:200 dilution) and incubated for 1 h in the dark. They were then washed three times with PBS and treated with 6% *Pisum sativum* agglutinin fluorescein isothiocyanate (FITC-PSA) for 1 h in the dark. The slides were then washed three times with PBS and a coverslip was placed on the sperm smear, after which the samples were observed using a Zeiss axiophot microscope (Carl Zeiss Inc., Thornwood, NY, USA).

### Immunofluorescence of zona-free eggs

For rLYZL6 localization, freshly prepared zona-free hamster eggs were incubated with 10 μg/mL rLYZL6 in 3% BSA/PBS at room temperature for 1 h. After three washes with 3% BSA/PBS, the eggs were incubated with anti-LYZL6 serum (1:100 dilution) for 30 min at room temperature. The eggs were then washed three times and incubated with fluorescein isothiocyanate-conjugated goat anti-rabbit IgG (1:200 dilution; Sigma-Aldrich) in the dark for 30 min at room temperature. After washing an additional five times, the eggs were mounted on slides and observed by phase contrast and fluorescence microscopy using a confocal laser-scanning microscope (FV-300; Olympus, Tokyo, Japan).

### Zona-free HEPT

The assay was performed as described [14]. Briefly, fresh human semen was mixed with an equal volume of TEST yolk buffer (12% v/v glycerol, 20% egg yolk, 10 μg/mL gentamicin sulfate; Irvine Scientific), incubated for 10 min at 37°C, cooled to 25°C and held at 4°C overnight. The cells were allowed to settle for ~24 h and were then washed and resuspended in Biggers, Whitten, and Whittingham (BWW) medium. The prepared spermatozoa were capacitated at a concentration of 20 × 10^6^ spermatozoa/mL for 5 h in BWW medium followed by 30 min incubation in calcium ionophore A23187 (Sigma-Aldrich). The capacitated spermatozoa were preincubated with anti-LYZL6 serum at different dilution at 37°C under 6% CO_2_ in humidified air for 1 h. Preimmune rabbit serum was used as a control. The top 100 μL was collected from each tube, washed with BWW medium and resuspended, giving a final concentration of 2 × 10^6^ spermatozoa/mL. Standard procedures were utilized for the collection of hamster eggs [14]. Freshly prepared zona-free eggs (~25 eggs per 100-μL drop) were added to suspensions of spermatozoa and co-incubated for 3 h. The eggs were washed three times in drops of BWW to remove loosely bound sperm and mounted on slides. To score binding and fusion, eggs were flattened and fixed with 2–5% glutaraldehyde for 1 min, ethyl alcohol 95% for 1 min and stained with acetolacmoid (Sigma-Aldrich) for 1 min. They were then examined using phase-contrast microscopy. A swelling sperm head was the criterion for fertilization. The numbers of attached spermatozoa and of swollen sperm heads in each egg were counted. The significance of any differences in means between the antiserum-treated groups and control was determined using Student’s *t* test. Value of *P* less than 0.05 was considered to be statistically significant.

## Results

### LYZL6 is detected in the human testis, epididymis and spermatozoa

**Fig 1:**
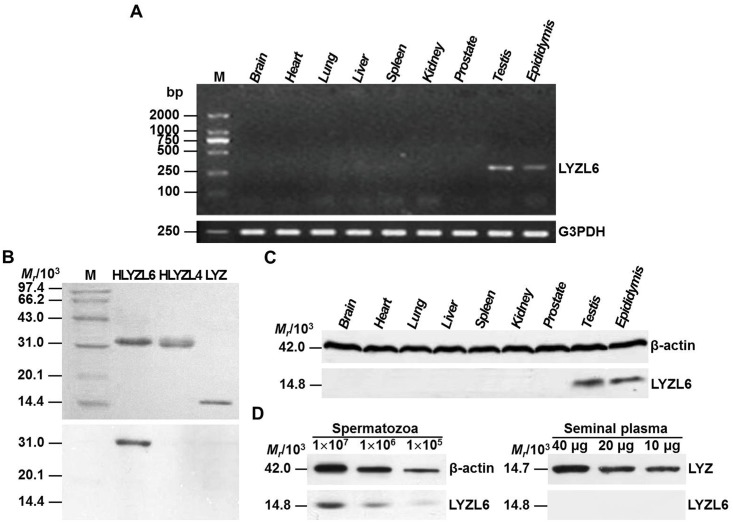
Tissue expression patterns of LYZL6. A) RT–PCR analysis. Lane M: DL2000 DNA marker. The expected sizes of PCR products were 296 bp for LYZL6 and 247 bp for G3PDH. Totally 14 tissues were examined. Nine of them were shown and the rest included the pancreas, small intestine, colon, thymus and skeletal muscle. B) Anti-LYZL6 serum specificity analysis. Top panel: SDS-PAGE; Bottom panel: western blotting. Lane M: molecular weight (MW) marker; The expected MW was ~14.7 kDa for LYZ and ~32.0 kDa for His-tagged LYZL6 (HLYZL6) and His-tagged LYZL4 (HLYZL4), which the extra MW resulted from Trx-tag, S-tag and restriction enzyme site of the pET32a vector. The amount of His-tagged LYZL6 and His-tagged LYZL4 loaded was 0.5 μg and that of LYZ was 0.1 μg. C) Western blot analysis of human tissue extracts. A band corresponding to full-length LYZL6 was evident in the testis and epididymis. No expression was detected in the other human tissues. D) Western blot analysis of LYZL6 in human semen. LYZL6 was identified in protein lysates representing different numbers of ejaculated spermatozoa (left), but not in seminal plasma (right). β-actin and LYZ were used as internal controls respectively.

Here we detected the tissue expression patterns of the *LYZL6* mRNA in different human tissues by RT-PCR. The results revealed that the *LYZL6* gene was selectively expressed in the testis and epididymis, but not in other human tissues (Fig 1A). Because of the high sequence similarity (42.0%) between LYZL6 and LYZL4, cross reactivity of anti-LYZL6 serum was checked with recombinant LYZL4 expressed in *E*. *coli*. No cross reactivity was observed with His-tagged LYZL4 and intact LYZ (Fig 1B). The presence of LYZL6 in the testis and epididymis was further confirmed by western blotting (Fig 1C). A single band of ~14.8 kDa, compatible with the predicted size of LYZL6, was detected in sperm extracts, but not in seminal plasma of human semen (Fig 1D).

### The presence of LYZL6 in human sperm extracts

**Fig 2:**
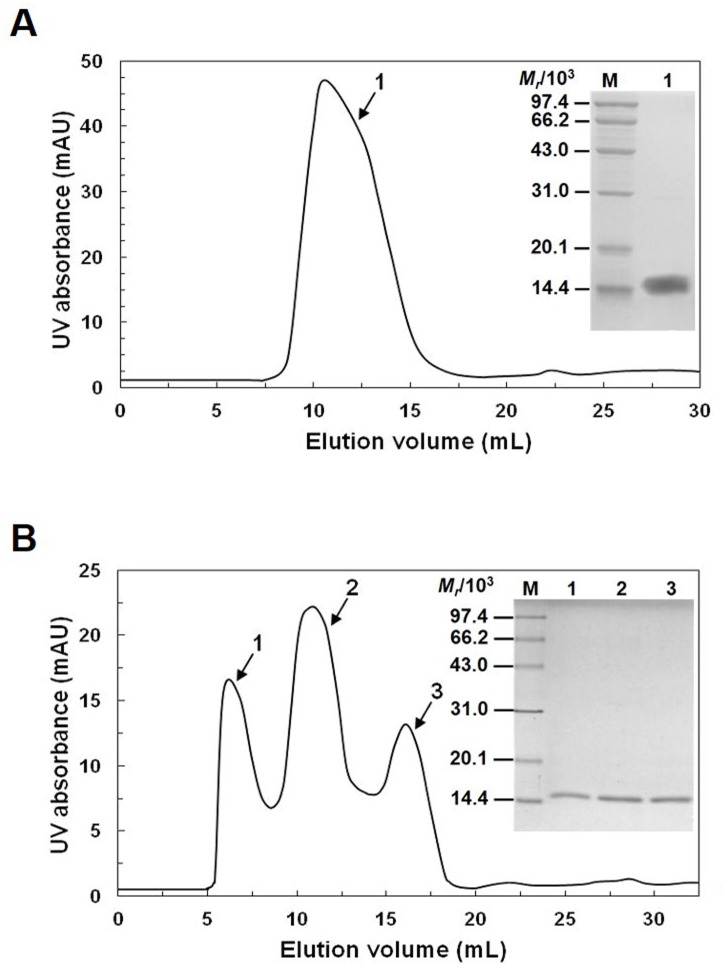
Isolation of native LYZL6 from human sperm extracts. A) Elution profile of chitin affinity chromatography. Inset: SDS–PAGE of the chitin column peak. Lane M: MW marker; Lane 1: peak 1. B) Elution profile of anion exchange chromatography. Inset: SDS–PAGE of the HiTrap Q column peaks. Lane M: MW marker; Lane 1–3: peak 1, 2, and 3. Bands in the gel were identified by MS.

To purify native LYZL6 protein from human sperm extracts, chitin affinity chromatography was utilized. As chitin contains β-1,4-linked N-acetylglucosamine units in its structure that can bind proteins of the c-type LYZ/α-lactalbumin family, it can be directly used for affinity purification without further chemical modification. By using this method, the LYZLs bound to spermatozoa were isolated and a single peak was observed (Fig 2A). Those negative-charged LYZLs were further separated by anion exchange chromatography and three elution peaks were observed (Fig 2B). To identify native LYZL6, we performed one-dimensional SDS–PAGE in conjunction with MALDI-TOF/TOF MS. The bands corresponding to three peaks were excised and subjected to MS analysis, and a lysozyme like molecule was identified in each peak. After the Mascot database search, the protein in the second elution peak was identified as LYZL6 with mascot score 79 and sequence coverage of 21% of the whole amino acid sequence of LYZL6, while that in the third peak was identified as SLLP1. Table 2 summarized the peptide sequences and search scores for the identified proteins.

**Table 2:** The identified LYZLs by MALDI-TOF/TOF MS.

Band number	Protein name with accession number	Fragment	Identified peptides	% Coverage	MASCOT score^a^	Observed Mr	Experimental Mr	Calculated Mr
2	Lysozyme-like protein 6 precursor [Homo sapiens] NP_001186880	97–102	IVSGAR	21	79	602.6123	601.5824	601.5709
		97–111	IVSGARGMNNWVEWR			1774.5152	1773.5217	1773.5337
		103–111	GMNNWVEWR			1192.3367	1191.3213	1191.3068
		112–127	LHCSGRPLFYWLTGCR			1910.1334	1909.1238	1909.1165
3	Sperm acrosome membrane-associated protein 3 isoform 1 [Homo sapiens] NP_776246	1–9	LYGRCELAR	40	112	1081.2319	1080.1176	1080.1324
		5–20	CELARVLHDFGLDGYR			1864.8750	1863.9631	1863.9532
		10–20	VLHDFGLDGYR			1292.3252	1291.2735	1291.2957
		62–75	WCSNLTPNVPNVCR			1601.8584	1600.8329	1600.8035
		76–87	MYCSDLLNPNLK			1411.6814	1410.5593	1410.5385
		76–95	MYCSDLLNPNLKDTVICAMK			2273.5930	2272.5756	2272.5723
		88–95	DTVICAMK			880.1236	880.0812	880.0653
		96–110	ITQEPQGLGYWEAWR			1834.0387	1833.8671	1833.8727
		111–116	HHCQGK			708.8941	708.8028	708.7948
		111–127	HHCQGKDLTEWVDGCDF			1991.1323	1990.1255	1990.1041
		117–127	DLTEWVDGCDF			1299.3827	1299.3424	1299.3332

^a^ Proteins with scores >66 are significant (P < 0.05).

### LYZL6 exhibits *in vitro* bacteriolytic activity against *M*. *lysodeikticus*

**Fig 3:**
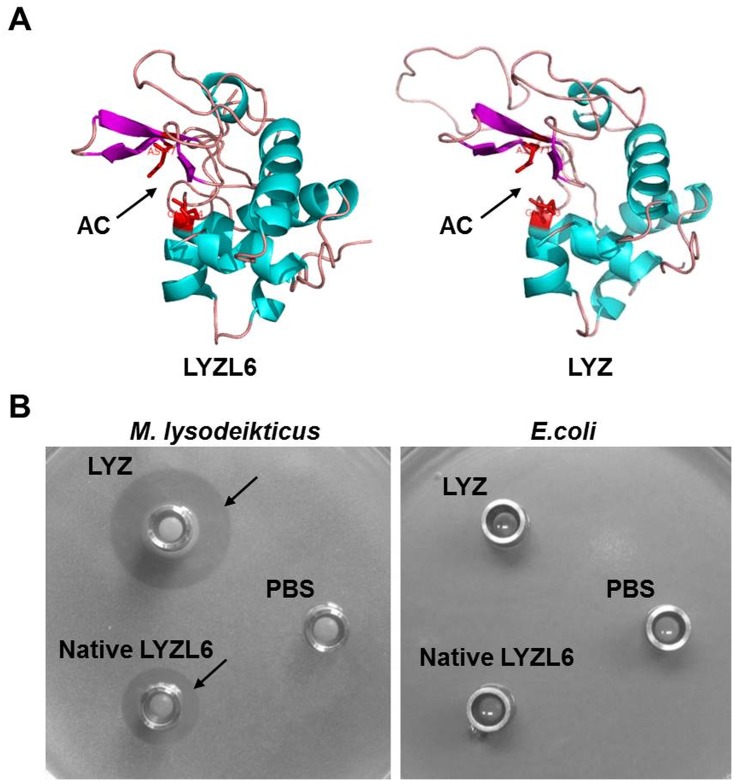
Bacteriolytic activity of LYZL6. A) Homology modeling of LYZL6 and LYZ. The structures of LYZL6 and LYZ were predicted using MODELLER 9.5. The active centers (AC) of LYZL6 and LYZ were shown (arrows). B) LYZL6 displayed bacteriolytic activity specifically against *M*. *lysodeikticus* but not *E*. *coli*. Bacteriolytic activity of LYZL6 was assessed using radial diffusion assay. Native LYZL6 in PBS (pH 6.0) was added to wells and the zone of clearance was observed using *M*. *lysodeikticus* (arrow), but not using *E*. *coli* as substrate. LYZ was used as a positive control and PBS was used as a negative control.

Homology modeling revealed a close structural similarity of the active center of LYZL6 with LYZ (Fig 3A). The RMSD estimated for backbone atoms of LYZL6 and LYZ was 0.69 which was within the agreeable limit. The bacteriolytic activity of native LYZL6 was tested using radial diffusion assay and two bacteria were used as substrates. LYZL6 showed enzymatic activity against model bacterium *M*. *lysodeikticus* (zone of clearance). However, no obvious bacteriolytic activity was observed against *E*. *coli* (Fig 3B).

### Separation of rLYZL6 by chitin-affinity chromatography

**Fig 4:**
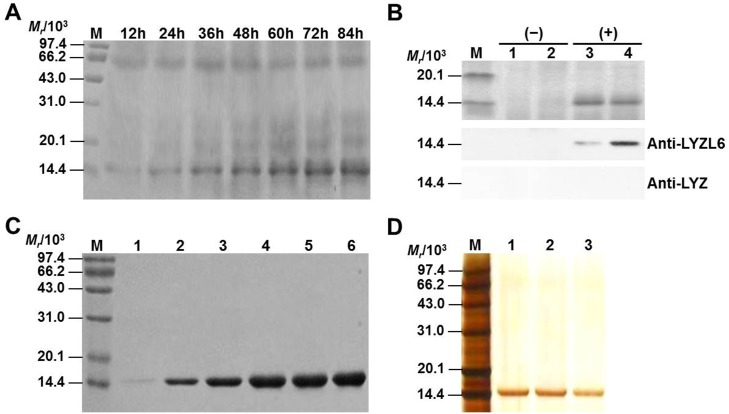
Identification of rLYZL6 expressed in *P*. *pastoris*. A) SDS–PAGE of LYZL6 expression at various time points after methanol induction. Lane M: MW marker. Supernatant protein (~10 μg per lane) was loaded. B) Western blot analysis of the fermentation supernatant. Lane M: MW marker; Lane 1–2: fermentation supernatant from un-transformed yeast; Lane 3–4: fermentation supernatant from recombinant yeast. C) SDS–PAGE of fractions collected by chitin affinity chromatography. The amount of rLYZL6 loaded was 0.1 μg (lane 1), 0.5 μg (lane 2), 1 μg (lane 3), 2 μg (lane 4–6). D) Silver stained SDS–PAGE of samples collected through size exclusion chromatography steps. Lane M: MW marker; Lane 1–3: rLYZL6 (~0.5 μg per lane). The samples were obtained from three separate experiments.

To determine the enzymatic properties of LYZL6, LYZL6 cDNA minus the signal sequence was fused to a tag-free vector to avoid being affected by artifacts and the processed form of the molecule produced in the methylotrophic yeast, *P*. *pastoris*, was directly secreted into the medium. Protein secretion in the culture broth was detected as early as 12 h after methanol induction and a band of ~14.8 kDa was apparent on a SDS–PAGE gel (Fig 4A). No endogenous LYZ or LYZL6 was identified by western blotting (Fig 4B). The rLYZL6 protein was also purified from the fermentation supernatant utilizing chitin-affinity chromatography to eliminate any contaminating proteins (Fig 4C and 4D). We generated dozens of milligrams of pure rLYZL6 protein for the subsequent analysis.

### The rLYZL6 protein activity is dependent on pH and Na^+^

**Fig 5:**
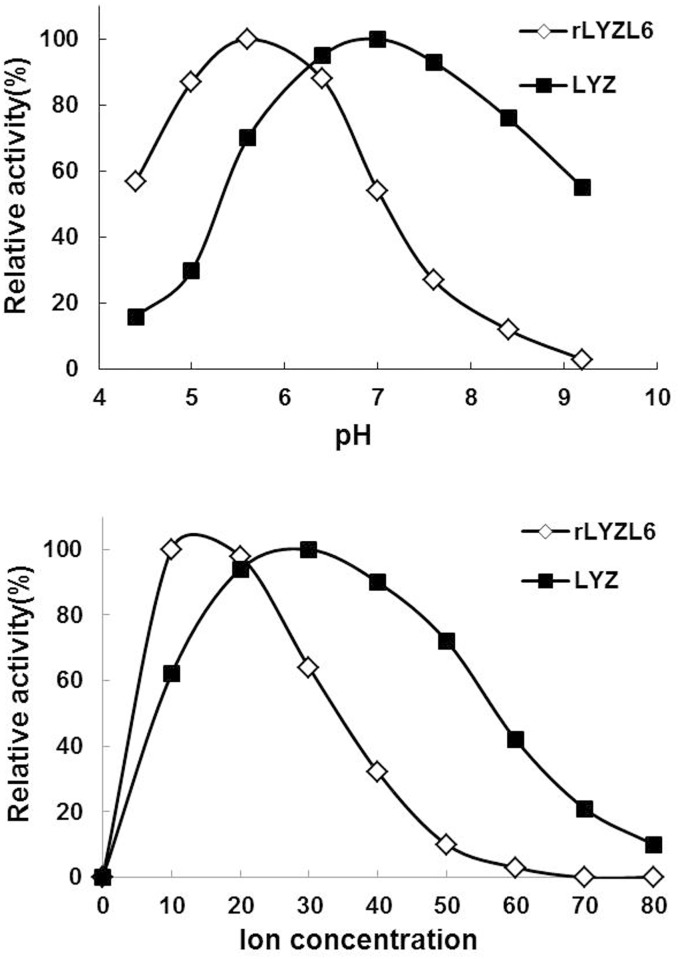
Effect of pH and ion concentration on the activity of rLYZL6. A) Activities of rLYZL6 and LYZ at different pH values, with a maximum activity at pH ~5.6. B) Effect of Na^+^ ion concentration on activities of rLYZL6 and LYZ. For all of the assays, rLYZL6 solution was added to substrate suspension and identical amounts of human milk LYZ were used as controls. All values have been normalized to the peak activity for each curve (100%).

Bacteriolytic activity of rLYZL6 displayed a pH dependence as indicated in Fig 5A, with a maximum at around pH 5.6. Bacteriolytic activity of rLYZL6 was also dependent on Na^+^ concentration (at pH 5.6), with a narrow ion concentration range and a peak activity at 15 mM Na^+^ concentration (Fig 5B). This is different from LYZ that shows the highest activity at around pH 7.0 in the presence of 30 mM Na^+^ and a relatively broad ion concentration range.

### The rLYZL6 protein shows weak bacteriolytic activity against Gram-positive bacteria at physiological pH

**Fig 6:**
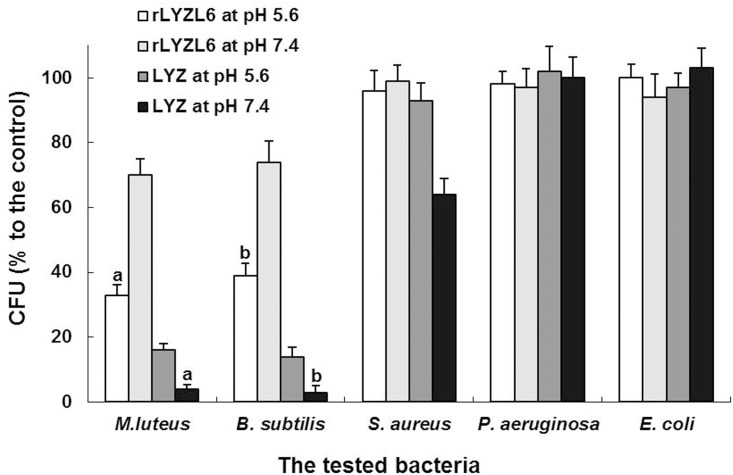
Bacteriolytic activities of rLYZL6 or LYZ under different conditions. The results are presented as the percentage of the control incubated without protein. The rLYZL6 protein showed high bacteriolytic activities against *M*. *lysodeikticus*, *and B*. *subtilis*. The growth inhibition caused by rLYZL6 decreased significantly after transferring to a high pH (*P* < 0.001). The rLYZL6 protein exhibited weaker bacteriolytic activities than LYZ against the sensitive strains (*P* < 0.05). The letters (a and b) were used to indicate the comparisons.

Reportedly, LYZ has membrane-lytic activity against Gram-positive bacteria. The bacteriolytic activities of rLYZL6 under different pH conditions were compared with the CFU assay. We found that both *M*. *lysodeikticus* and *B*. *subtilis*, but not the other three strains, were highly susceptible to rLYZL6. The bacteriolytic activities of rLYZL6 decreased significantly when pH was elevated from 5.6 to 7.4 (*P* < 0.001). By contrast, bacteriolytic activities of LYZ increased significantly when pH was elevated from 5.6 to 7.4. In the meantime, the bacteriolytic activities of rLYZL6 against sensitive bacterial strains were significantly lower than those of LYZ (*P* < 0.05; Fig 6).

### LYZL6 is located on the postacrosomal region of human spermatozoa and rLYZL6 can bind to the egg plasma membrane

**Fig 7:**
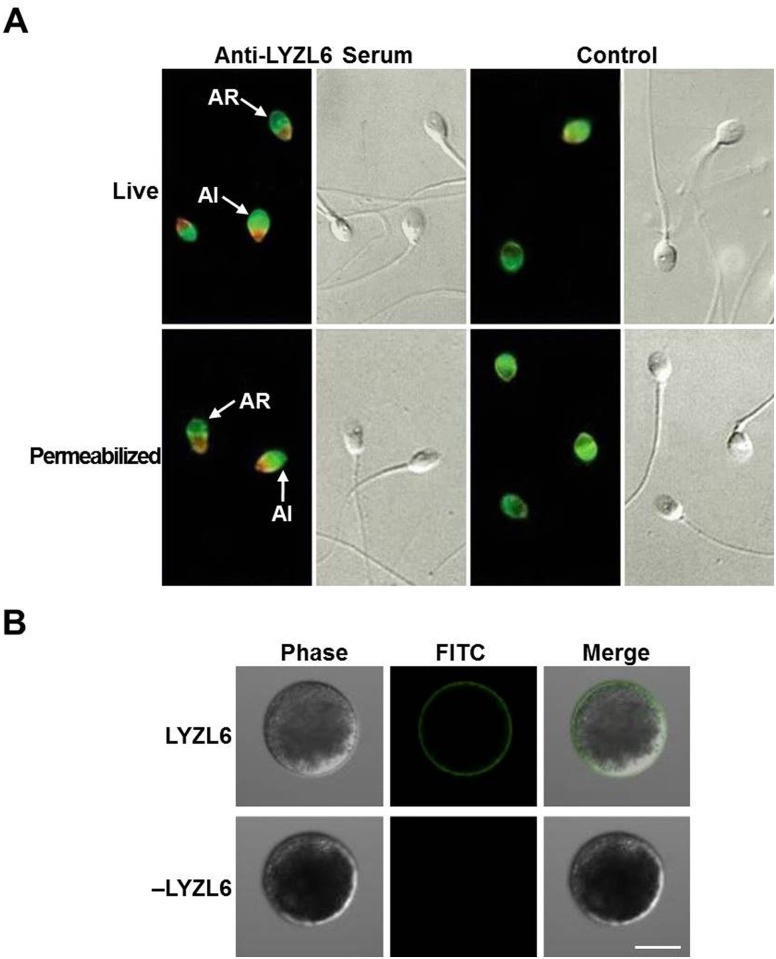
Immunofluorescence staining of LYZL6 in spermatozoa and rLYZL6 bound to zona-free hamster eggs. A) Live and permeabilized spermatozoa were incubated with the anti-LYZL6 serum or preimmune rabbit serum, followed by a rhodamine-conjugated goat anti-rabbit secondary antibody. A band-like pattern (red) was detected on the postacrosomal region for LYZL6 in acrosome-reacted (AR) and in acrosome intact (AI) cells. No similar staining of LYZL6 was seen in the control cells. The FITC-PSA staining was used to distinguish membrane changes in spermatozoa due to acrosome reaction. B) The eggs were incubated with rLYZL6 (upper panels) or without rLYZL6 (lower panels) and probed with the anti-LYZL6 serum. Scale bar = 50 μm.

Studies were next carried out to determine whether LYZL6 was present on the sperm cell surface. Immunofluorescent staining was used to investigate the subcellular localization of LYZL6 on live and permeabilized spermatozoa. LYZL6 immunostaining was detected predominantly on the postacrosomal region of 90% live cells, and its localization appeared to be unrelated to acrosome status. This was further consolidated by using permeabilized spermatozoa. Control preimmune serum produced little staining (Fig 7). Altogether, these data indicate that LYZL6 is located on the postacrosomal membrane, but not in the postacrosomal cytoplasm. To determine if rLYZL6 bound to the egg plasma membrane, zone-free hamster eggs were incubated with rLYZL6, washed, and probed with the anti-LYZL6 serum. Significant staining was observed after rLYZL6 labelling, but no staining occurred in the control cells (Fig 7B).

### Anti-LYZL6 serum decreases the numbers of spermatozoa fused per hamster egg *in vitro*

**Fig 8:**
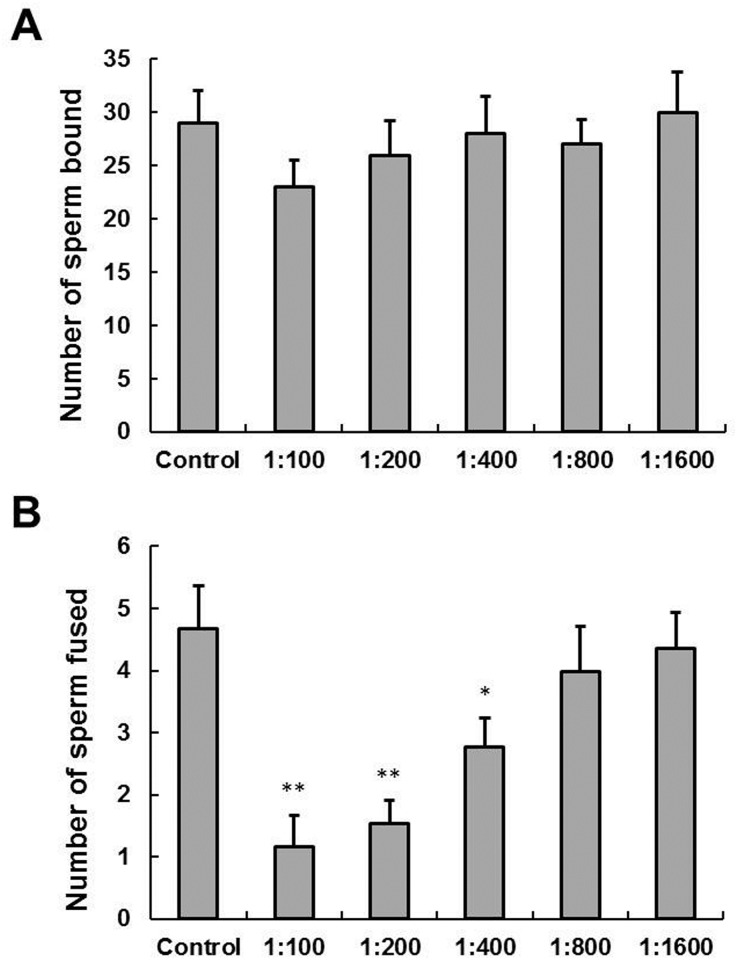
Effect of LYZL6 antiserum on sperm-egg binding and fusion. A) The mean number (± SD) of spermatozoa bound per egg did not differ significantly between the control and the antiserum-treated groups (control: 29 ± 3.07; anti-LYZL6 serum: 1: 100, 23 ± 2.48; 1: 200, 26 ± 3.20; 1: 400, 28 ± 3.56; 1: 800, 27 ± 2.34; 1: 1600, 30 ± 3.85). B) The mean number of spermatozoa fused per egg decreased significantly following incubation of spermatozoa with higher antiserum concentrations (control: 4.68 ± 0.69; anti-LYZL6 serum: 1: 100, 1.16 ± 0.51; 1: 200, 1.54 ± 0.36; 1: 400, 2.77 ± 0.46; 1: 800, 3.98 ± 0.73; 1: 1600, 4.35 ± 0.59). *P < 0.05; **P < 0.01.

The zona-free HEPT was performed to explore the role of LYZL6 in human sperm function. The capacitated human spermatozoa were preincubated with different dilution of anti-LYZL6 serum to block LYZL6 in spermatozoa and then were coincubated with zona-free hamster eggs. Although there was a decrease in the number of spermatozoa attached per egg after prior incubation with 1: 100 dilution of anti-LYZL6 serum (23 per egg), the mean number of spermatozoa was not significantly different from the control group (29 per egg; *P* ≤ 0.2; Fig 8A). By contrast, at 1: 400, 1: 200 and 1: 100 dilution, anti-LYZL6 serum caused a decrease in the number of spermatozoa fused per egg by 43%, 62% and 71%, which was statistically significant when compared with the control group (anti-LYZL6 serum, 1: 400, 2.67 per egg; *P* < 0.05; 1: 200, 1.78 per egg; 1: 100, 1.36 per egg; *P* < 0.01; control, 4.68 per egg; Fig 8B).

## Discussion

To understand the function of LYZL6, we initially studied tissue expression patterns of *LYZL6* mRNA and LYZL6 protein in different human tissues. As reported, both e *LYZL6* mRNA and LYZL6 protein were found in the testis and epididymis [6]. The bioinformatics analysis has shown that LYZL6 has a signal peptide on the N-terminus of its amino acid sequence, suggesting that it may be secreted from the cells to exhibit bacteriolytic activity. We questioned whether LYZL6 was related to spermatozoa like SLLP1 and mouse LYZL4 and whether it existed in seminal plasma. We found that LYZL6 was present in sperm extracts, but not obviously in seminal plasma.

LYZL6 has an amino acid sequence similar to LYZ and conserves several key molecular features, including retention of putative substrate-binding and two essential catalytic residues (Glu35 and Asp52), which are able to catalyze the hydrolysis of β-1,4-linked glycosidic bonds between N-acetylmuramic acid and N-acetylglucosamine in the peptidoglycan of bacterial cell walls. It is likely that LYZL6 retains catalytic activity, yet to a lesser degree. To isolate LYZL6 from sperm extracts, we performed chitin affinity chromatography in combination with anion exchange chromatography. By using this strategy, several LYZLs were isolated and those negative-charged were further separated on a HiTrap Q column. The presence of LYZL6 was subsequently identified by MS. LYZL6 was predicted to undergo some post-translational modification such as glycosylation, which might result in low sequence coverage from the MS data. Due to chitin also containing β-1,4-linked N-acetylglucosamine, it is inferred that the conformation of the substrate-binding sites of LYZL6 is similar to that of LYZ. As expected, LYZL6 exhibited *in vitro* bacteriolytic activity against *M*. *lysodeikticus*.

In the male reproduction tract, there are a few peptides and proteins secreted by the testis and/or epididymis that attach to maturing spermatozoa. These molecules such as hCAP-18/SOB3, β-defensin and EPPIN exhibit potent antibacterial activity, thereby forming important components of innate immunity [15–18]. For example, hCAP-18/SOB3 secreted by the epididymal epithelium is localized in human spermatozoa and plays an antibacterial role [15, 16]. All of these peptides and proteins are cationic under normal physiological conditions, with spatially separated hydrophobic and charged regions, which is crucial to their bacteriolytic mechanisms of action on pathogens. In prokaryote membranes, there are many highly anionic phospholipids orientated outward. They interact with positively charged antibacterial molecules and are bactericidal via a variety of mechanisms including membrane depolarization, membrane permeabilization, induction of hydrolases, and disruption of membrane functions [19]. Because LYZL6, with a predicted isoelectric point of 5.44 is negatively charged in seminal plasma, this may result in a decrease in its bacteriolytic activity. In humans, LYZ is synthesized in the secretory cells of a variety of exocrine glands and then secreted in their respective body fluids. However, two earlier systematic investigations suggested that LYZ was not expressed in testicular tissues [20, 21]. Therefore LYZL6 might play a role similar to LYZ in the innate immune defense of the human male reproductive tract.

To further investigate the role of LYZL6, we prepared a recombinant version using *P*. *pastoris*. In the light of the advantages of higher eukaryotic systems such as similar chaperones to humans and post-translational modifications, we employed this yeast as the expression host for this eukaryotic protein. As expected, the samples of fermentation supernatants exhibited bacteriolytic activity against *M*. *lysodeikticus*, suggesting the successful secretion of this recombinant protein. The rLYZL6 protein was also purified utilizing chitin affinity chromatography. We conducted a series of studies to determine its enzymatic properties and found that when using *M*. *lysodeikticus* as an initial substrate, rLYZL6 reached a peak bacteriolytic activity of 1.57 × 10^4^ U/mg at pH 5.6 and a Na^+^ concentration of 15 mM, about 21.1% of the activity of LYZ (equivalent to 7.45 × 10^4^ U/mg at pH 7.0 and a Na^+^ concentration of 30 mM). In addition, the bacteriolytic activity of rLYZL6 was significantly lower than LYZ for sensitive strains in various conditions. It is noteworthy that at pH 7.4, which is within the normal range of human seminal plasma (pH 7.2–7.6), rLYZL6 had only 33% of its peak activity and exhibited relatively weak bacteriolytic activity. Hence, rLYZL6 seems not to be able to efficiently fight against virulent microbes *in vivo*, inferring that LYZL6 does not mainly function as a defense factor in the innate immunity of the male reproductive tract. However, it cannot exclude the possibility i that LYZL6 might have a bacteriolytic role in the acidic environment of the female reproductive tract.

In the mammalian testis, undifferentiated germ cells transform into spermatozoa through a complex series of events. The ability to fertilize eggs requires many proteins secreted by testicular cells, such as SLLP1, one member of the c-type lysozyme/α-lactalbumin family. Immunofluorescence staining of ejaculated human spermatozoa showed LYZL6 protein was localized on the postacrosomal membrane. This is consistent with previous studies showing that mouse LYZL6 is detected on the post-acrosomal region and the midpiece of mature epididymal spermatozoa [10]. We predict that LYZL6 might be secreted by the male reproductive tract epithelium, and then becomes concentrated on spermatozoa. Although SLLP1 is located on the acrosomal region of human spermatozoa [8], mouse LYZL4 is present on the acrosomal region and principal piece of the sperm tail [7]. The discrepancy between the subcellular localization of LYZLs suggests that these homologous proteins might have different functions in spermatozoa.

For successful fertilization, spermatozoa undergo a complex series of steps, including capacitation, penetrating the cumulus, binding to the zona pellucida (ZP), undergoing acrosomal reaction, penetrating the ZP, attaching to the oolemma, undergoing sperm–oocyte membrane fusion, penetrating the ooplasm and participating in activation of the oocyte [22–24]. Using the zona-free HEPT, there was no significant decrease in the number of spermatozoa attached per egg. However, immunoneutralization of LYZL6 with its antisera significantly reduced the number of spermatozoa fused per egg in a dose-dependent manner, whereas anti-SLLP1 serum significantly decreased the number of spermatozoa bound per egg [8]. This suggests a different role of LYZL6 from SLLP1. SLLP1 is found in the acrosomal matrix where it interacts with a binding partner, SAS1B, located in the egg plasma membrane [9]. Labeling of zona-free eggs with rLYZL6 also revealed a significant staining in the egg plasma membrane although it displays a different localization from SLLP1. This suggests that a different binding partner may interact with LYZL6 during sperm–egg fusion. In human and mouse SLLP1, double changes to the catalytic sites result in a complete loss of the hydrolyzing activity against β-1,4-linked glycosidic bonds [8, 25]. Mouse and rat LYZL4 also do not exhibit any hydrolyzing activity because of the replacement of aspartate by glycine at one catalytic site [7, 26]. Therefore, we cannot eliminate the possibility that human LYZL6 might participate in fertilization by another mechanism.

## Conclusions

Although LYZL6 shows distinct enzymatic properties compared with LYZ, it exhibits a much lower bacteriolytic activity under physiological conditions. However, anti-LYZL6 serum significantly decreases the number of spermatozoa fused per hamster egg *in vitro*, suggesting an important role in fertilization which is different from the antibacterial one suggested by a previous study [10]. Based on our findings, we plan to identify the binding partner in the egg plasma membrane for LYZL6 and further elucidate the molecular mechanisms by which LYZL6 contributes to fertilization.

## Supporting information

S1 Fig Alignment of amino acid sequences of human c−type lysozyme family. Gaps that were introduced into the sequences were shown as dashes; Residues that were shaded showed the signal peptide and eight conserved cysteine residues; Inverted triangles indicated the key amino acids involved in catalytic activity.
(TIF)

S2 Fig SDS-PAGE of purified recombinant LYZL6. Lane 1: Negative control; Lane 2, 3: Purified recombinant LYZL6; Lane 4: Fermentation supernatant; Lane M: Molecular weight marker.
(TIF)

S3 Fig The RT-PCR detection of the LYZL6 mRNA. Lane M: DL2000 DNA marker. The expected sizes of PCR products were 296 bp for LYZL6 and 247 bp for G3PDH. The LYZL6 mRNA was detected in the testicular and epididymal cDNA library, but not in the sperm cDNA library, suggesting a testicular and epididymal tissue origin.
(TIF)

S4 Fig HPLC of purified native LYZL6. (TIF)

S5 Fig Immunolocalization of LYZL6 in human testis. A) DAB staining was observed in late-stage spermatocytes and round spermatids, but not in other germ or tissue cells in the testis; B) Pre-immune rabbit serum was used as a control.
(TIF)

S6 Fig Western blot analysis of LYZL6 in spermatozoa before and after capacitation. LYZL6 was identified in protein lysates of spermatozoa before and after capacitation. There was not an obvious decrease in the amount of LYZL6. β-actin was used as an internal control.
(TIF)

S1 Table Sequence identity between LYZLs (%). (DOCX)
