# Supplementary figures and images for: LYZL6, an acidic, bacteriolytic, human sperm-related protein, plays a role in fertilization
Source: PLoS One. 2017 Feb 9;12(2):e0171452. doi: 10.1371/journal.pone.0171452 (PMC5300149; doi:10.1371/journal.pone.0171452)

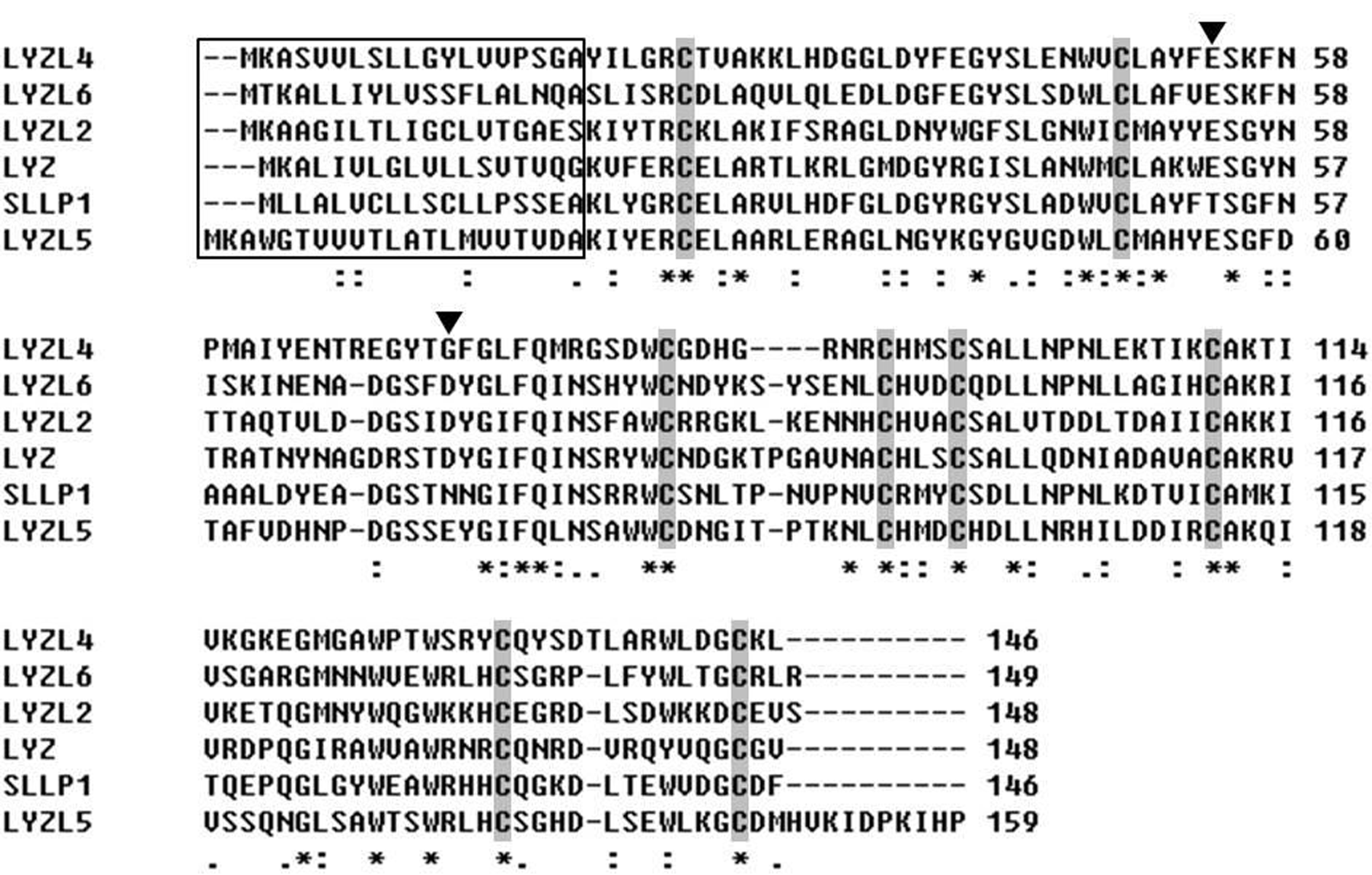

Supplement: S1 Fig — Gaps that were introduced into the sequences were shown as dashes; Residues that were shaded showed the signal peptide and eight conserved cysteine residues; Inverted triangles indicated the key amino acids involved in catalytic activity. (TIF) [file pone.0171452.s001.tif]

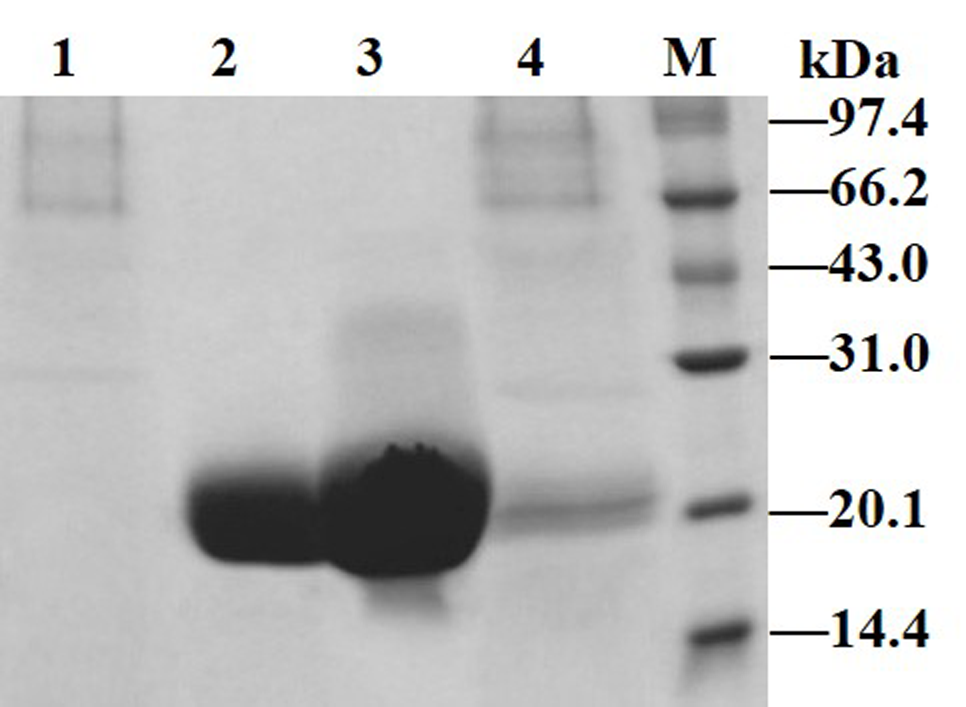

Supplement: S2 Fig — Lane 1: Negative control; Lane 2, 3: Purified recombinant LYZL6; Lane 4: Fermentation supernatant; Lane M: Molecular weight marker. (TIF) [file pone.0171452.s002.tif]

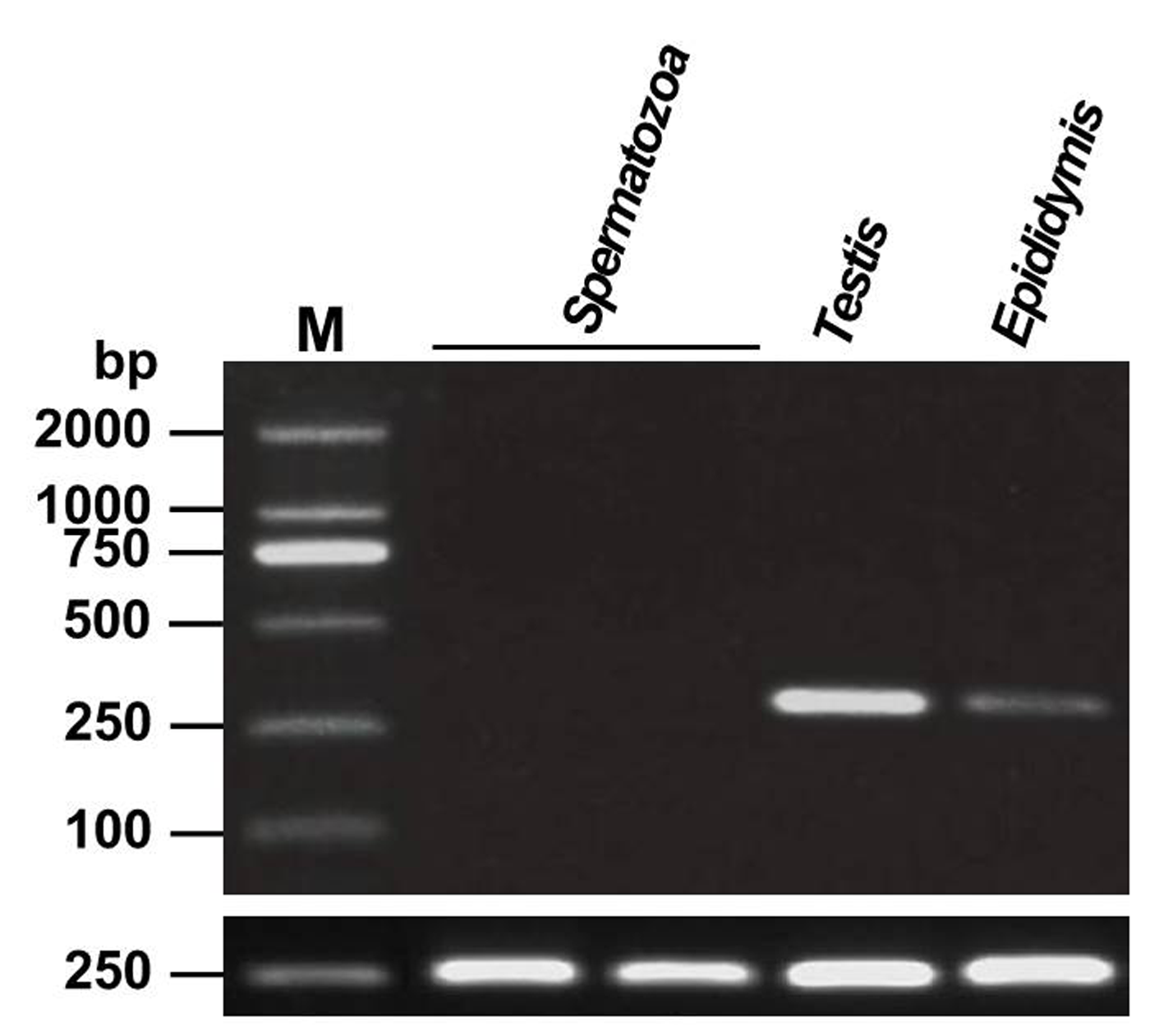

Supplement: S3 Fig — Lane M: DL2000 DNA marker. The expected sizes of PCR products were 296 bp for LYZL6 and 247 bp for G3PDH. The LYZL6 mRNA was detected in the testicular and epididymal cDNA library, but not in the sperm cDNA library, suggesting a testicular and epididymal tissue origin. (TIF) [file pone.0171452.s003.tif]

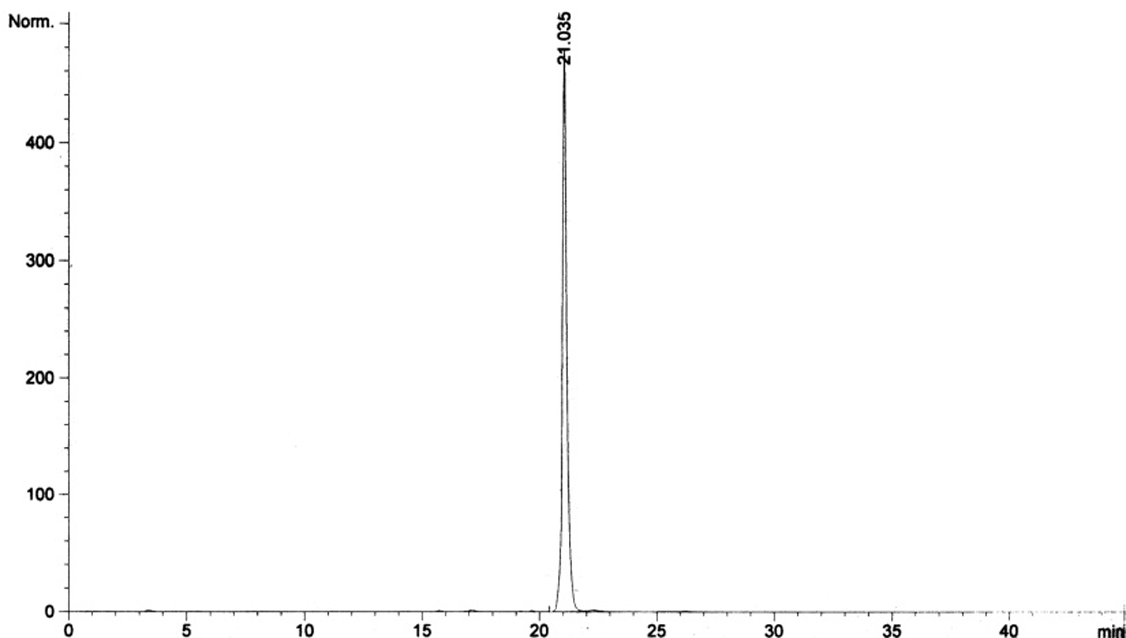

Supplement: S4 Fig — (TIF) [file pone.0171452.s004.tif]

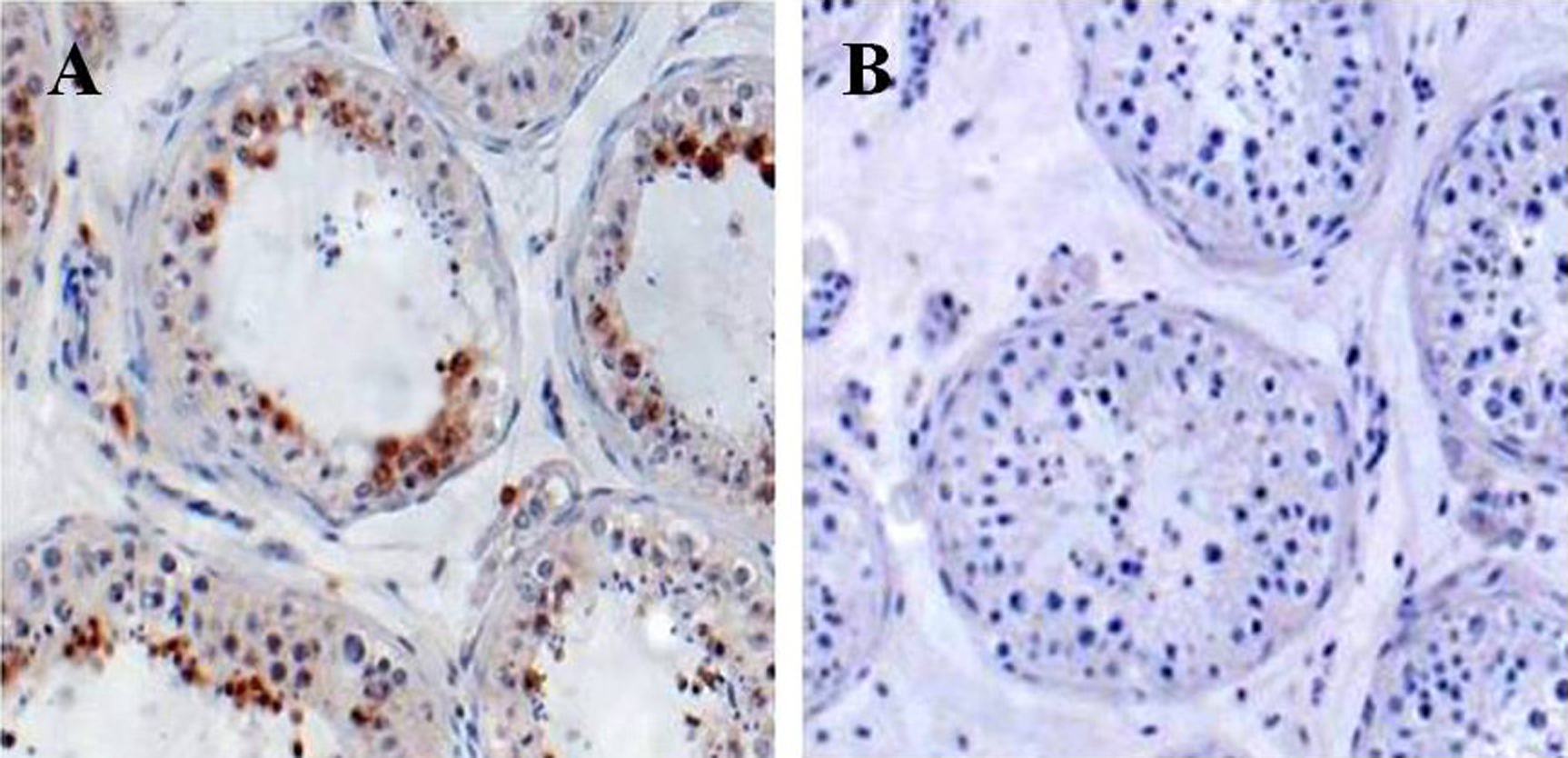

Supplement: S5 Fig — A) DAB staining was observed in late-stage spermatocytes and round spermatids, but not in other germ or tissue cells in the testis; B) Pre-immune rabbit serum was used as a control. (TIF) [file pone.0171452.s005.tif]

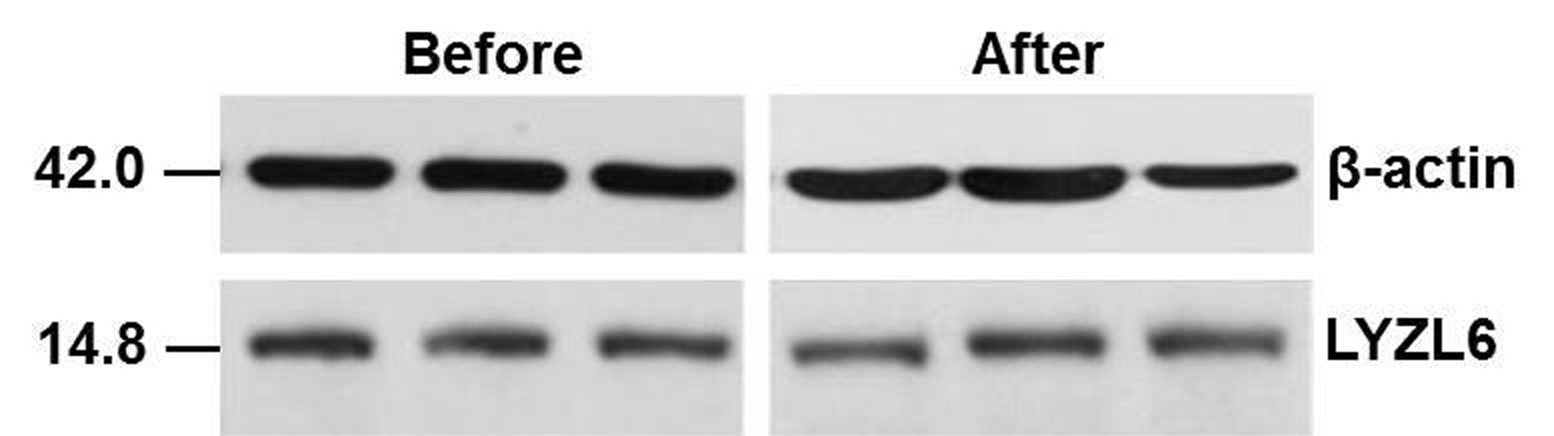

Supplement: S6 Fig — LYZL6 was identified in protein lysates of spermatozoa before and after capacitation. There was not an obvious decrease in the amount of LYZL6. β-actin was used as an internal control. (TIF) [file pone.0171452.s006.tif]
