# Supplementary material for: LYZL6, an acidic, bacteriolytic, human sperm-related protein, plays a role in fertilization
Source: PLoS One. 2017 Feb 9;12(2):e0171452. doi: 10.1371/journal.pone.0171452 (PMC5300149; doi:10.1371/journal.pone.0171452)
Supplement: S1 Table — (DOCX) [file pone.0171452.s007.docx]

**S1 Table Sequence Identity between LYZLs (%)**

|  | LYZL2 | SLLP1 | LYZL4 | LYZL5 | LYZL6 | LYZ |
| --- | --- | --- | --- | --- | --- | --- |
| LYZL2 |  | 41.9 | 38.0 | 42.1 | 38.5 | 45.3 |
| SLLP1 |  |  | 43.0 | 40.9 | 42.6 | 47.3 |
| LYZL4 |  |  |  | 32.9 | 42.0 | 38.0 |
| LYZL5 |  |  |  |  | 39.6 | 37.9 |
| LYZL6 |  |  |  |  |  | 40.7 |
| LYZ |  |  |  |  |  |  |
